# The emergence of SARS-CoV-2 variants threatens to decrease the efficacy of neutralizing antibodies and vaccines

**DOI:** 10.1042/BST20210859

**Published:** 2021-12-02

**Authors:** Kensaku Murano, Youjia Guo, Haruhiko Siomi

**Affiliations:** Department of Molecular Biology, Keio University School of Medicine, Tokyo, Japan

**Keywords:** antibodies, SARS-CoV-2, variant

## Abstract

The novel coronavirus severe acute respiratory syndrome coronavirus 2 (SARS-CoV-2) is the cause of the coronavirus disease (COVID-19) pandemic. As of August 2021, more than 200 million people have been infected with the virus and 4.3 million have lost their lives. Various monoclonal antibodies of human origin that neutralize the SARS-CoV-2 infection have been isolated from convalescent patients for therapeutic and prophylactic purposes. Several vaccines have been developed to restrict the spread of the virus and have been rapidly administered. However, the rollout of vaccines has coincided with the spread of variants of concern. Emerging variants of SARS-CoV-2 present new challenges for therapeutic antibodies and threaten the efficacy of current vaccines. Here, we review the problems faced by neutralizing antibodies and vaccines in the midst of the increasing spread of mutant viruses.

## Introduction

The outbreak of coronavirus disease (COVID-19) caused by the severe acute respiratory syndrome coronavirus 2 (SARS-CoV-2) has resulted in a global health emergency and economic disruption [1,2]. SARS-CoV-2 is an enveloped single-stranded RNA virus with a surface-anchored spike glycoprotein. Cellular entry of the virus is triggered by the binding of the receptor-binding domain (RBD) of the spike protein to the cellular receptor, human angiotensin-converting enzyme 2 (ACE2) [3,4]. The spike protein comprises a trimeric assembly of two subunits, S1 and S2. Host proteases, such as furin, cleave the spike protein at the S1/S2 site [5]. The S2 region drives the fusion of the viral and host cell membranes. Most neutralizing antibodies developed for therapies and prophylaxis target viral entry mediated by the spike-ACE2 interaction [6]. Neutralizing antibodies are potentially beneficial for populations vulnerable to other diseases. Along with the development of neutralizing antibodies, various types of vaccines (e.g. mRNA-based vaccines, protein-based vaccines, adenoviral vector vaccines, and a whole-virus inactivated vaccines) against SARS-CoV-2 have been developed faster than ever before, where to 46.1% of the world population has been administered at least one dose (https://ourworldindata.org/covid-vaccinations). Among them, mRNA-based vaccines have been developed and authorized for emergency use in less than a year after the onset of the pandemic. Vaccines developed against SARS-CoV-2, consist of synthetic mRNA encoding the spike protein, packaged in lipid nanoparticles to deliver mRNA to cells, and are ∼95% effective in preventing COVID-19 [7–10]. These vaccines prevent disease progression of in individuals as well as the transmission of infection (‘herd immunity') — this function prevents the population as a whole. However, these strategies for controlling the spread of COVID-19 are limited by the emergence of highly transmissible and virulent variants that escape neutralizing antibodies and vaccines. Therefore, it is crucial to continue surveying the virus evolution, and a great deal of effort has been put into this investigation to date (GISAID COVID-19 variant tracker; https://www.gisaid.org/hcov19-variants/). This review discusses the recent reports focusing on the effect of the emergence of viral variants on the use and efficacy of neutralizing antibodies and vaccines, highlighting the need to develop and improve them continuously.

## Isolation of antibodies against SARS-CoV-2

**Figure 1.**
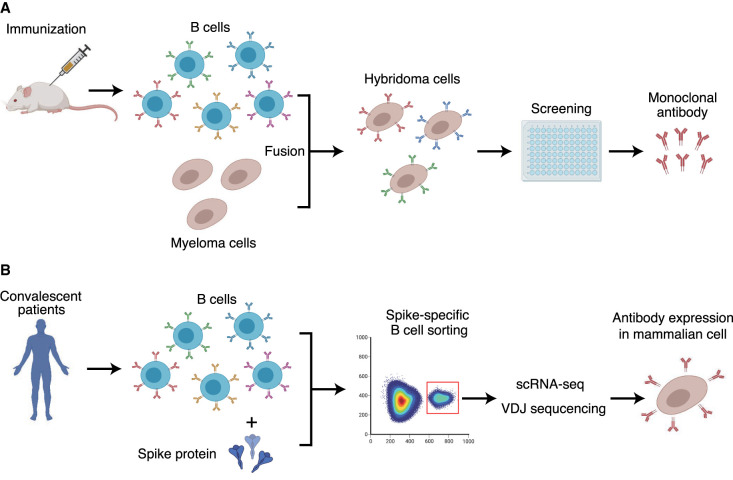
Generation of monoclonal antibodies. (**A**) The traditional hybridoma technology provides a reliable source of mouse monoclonal antibodies. The fusion of a B lymphocyte from an immunized mouse and an immortalized myeloma cell results in a hybridoma secreting monoclonal antibodies, potentiating natural cognate antibody pairing information compared with that obtained using recombinant antibodies. Mouse antibodies, if not humanized, are not suitable for clinical applications due to their immunogenicity. (**B**) Technology for the production of human recombinant antibodies. Spike-specific B cells from convalescent patients are single-cell sorted using flow cytometry, followed by determination of the antibody variable genes. The genes are cloned into human IgG expression vectors and expressed in mammalian cells such as HEK293T cells. Figures generated with BioRender (https://biorender.com/).

In early 2020, when COVID-19 began to spread, the isolation of multiple neutralizing monoclonal antibodies against SARS-CoV-2 was reported (Table 1) [6]. This section outlines the technologies used for antibody generation. The advent of hybridoma technology in 1975 provided a reliable source of mouse monoclonal antibodies [11]. Hybridomas are cloned cell lines produced by the fusion of B lymphocytes and immortalized myeloma cells and have both the antibody-producing ability of B cells and the longevity and reproductivity of myeloma cells (Figure 1A). SARS-CoV-1 hybridoma produces an antibody (47D11, which is later reformatted to a complete human immunoglobulin) that shows potent cross-neutralizing efficacy against both SARS-CoV-1 and SARS-CoV-2 [12,13]. Other mouse monoclonal antibodies (S1D7, S3D8, and R52) against the spike protein not only demonstrated neutralizing activity but also exhibited robust performance in immunoassays, including western blotting, enzyme-linked immunosorbent assay (ELISA), and immunofluorescence [14]. Monoclonal antibodies produced by hybridoma are secreted into the culture supernatant and preserve the natural cognate antibody pairing information. Their production is simple and low-cost, and their quality is stable. However, such antibodies will be useful for treatment only if they are chimeric or humanized, owing to their immunogenicity [15,16]. Traditional hybridoma technology is being replaced by human recombinant antibody production technology for the isolation of antibodies against SARS-CoV-2. The antibodies produced can be isolated and used in human therapeutics more efficiently.

**Table 1:** List of monoclonal neutralizing antibodies in this review

Antibody	Source	Neutralization (IC_50_)	Sponsors	Remarks	References
47D11	Mouse hybridoma	570 ng/ml		Immunization with SARS-CoV-1 Spike, cross-reactivity to SARS-CoV-2	[13]
S1D7	Mouse hybridoma	405.2 ng/ml		Available from RCB^1^, applicable for immunoprecipitation, immunofluorescence, ELISA	[14]
S3D8	Mouse hybridoma	139 ng/ml		Available from RCB^1^, applicable for immunoprecipitation, immunofluorescence, ELISA	[14]
R52	Mouse hybridoma	ND		Available from RCB^1^, applicable for western blotting	[14]
Casirivimab(REGN10933)	Convalescent patient/humanized mouse	37.4 pM	Regeneron	A cocktail of casirivimab and imdevimab, termed as REGN-COV2, receives EUA.	[42,44]
Imdevimab(REGN10987)	Convalescent patient/humanized mouse	42.1 pM	Regeneron	A cocktail of casirivimab and imdevimab, termed as REGN-COV2, receives EUA.	[42,44]
REGN10985	NA	NA	Regeneron	A combination of the antibody and REG-COV2 limits emergence of new escape variants.	[74]
Bamlanivimab(LY-CoV555/Ab169)	Convalescent patient	20–49 ng/ml	AbCellera and Eli Lilly and Company	A cocktail with etesevimab has EUA. FDA has revoked the EUA for bamlanivimab monotherapy.	[47,50,51]
Etesevimab (LY-CoV016/CB6)	Convalescent patient	36 ng/ml	Junshi Biosciences and Eli Lilly and Company	A cocktail of bamlanivimab and etesevimab has EUA.	[48,50]
Sotrovimab (VIR-7831/S309)	Convalescent patient	79 ng/ml	Vir Biotechnology Inc and GlaxoSmithKline	Sotrovimab has EUA.	[20,53]
PiN-21	Llama serum	22 pM		Inhalable nanobody, protect hamster at ultra-low dose.	[55]

An updated list of neutralizing antibodies under clinical studies can be found at COVID-19 Biologics Tracker (https://www.antibodysociety.org/covid-19-biologics-tracker/). Comprehensive list of neutralizing antibodies is summarized by Valdez-Cruz et al. [6].

1 RCB, Riken BRC Cell Bank (https://cell.brc.jp/en/).

The recent isolation of potent neutralizing antibodies has been driven by single-cell cloning technology (Figure 1B). These recombinant antibodies were derived from B cells of convalescent patients. Antigen-specific memory B cells were sorted using flow cytometry, and cognate variable regions of heavy and light chains were determined using reverse transcription polymerase chain reaction or single-cell 5′-mRNA and V(D)J sequencing [17]. In this method, variable regions were cloned into the human IgG expression vectors. Recombinant antibodies were secreted by HEK293 or CHO cells co-transfected with vectors. The process of antibody isolation has been dramatically shortened compared with that of the generation of traditional monoclonal antibodies via a hybridoma [18]. These human antibodies can circumvent the potential risks of human anti-mouse antibody responses and other side effects [16]. Although these monoclonal antibodies are recombinant, they are appropriate for direct use in humans as they are humanized. Therefore, most neutralizing antibodies against SARS-CoV-2 have been isolated from convalescent patients [6,19,20]. The use of high-throughput sequencing for profiling B-cell and T-cell receptors has resulted in a rapid increase in data generation. This adaptive immune receptor repertoire (AIRR)-seq data set is available through the AIRR Data Commons (https://www.antibodysociety.org/the-airr-community/airr-data-commons/).

## Passive immunization

Passive immunization can control the pandemic by providing immediate protection and complementing the development of prophylactic vaccines [21,22]. Passive immunization against infectious diseases can be traced back to the late 19th century and the work of Emil von Behring and Shibasaburo Kitasato on the serotherapy of tetanus and diphtheria [23]. They showed that serum from rabbits immunized with tetanus toxin could prevent tetanus in rabbits. Recent large outbreaks of viral diseases have renewed interest in convalescent plasma therapy (CPT). CPT has been used to treat many infectious diseases such as influenza [24,25], respiratory syncytial virus (RSV) infection [26], Middle East respiratory syndrome (MERS) [27], and SARS-CoV-1 [28]. As soon as the COVID-19 pandemic began, two research groups suggested that CPT could be a promising method for the treatment of COVID-19 [29,30]. The US Food and Drug Administration (FDA) and the European Center for Disease Control approved CPT as a therapeutic, and clinical trials have been conducted in many countries [31]. However, the scarcity of randomized controlled trials of CPT makes it difficult to assess the relative benefit-to-risk ratio of this strategy. Furthermore, convalescent plasma has a high titer of antibodies against the virus, which should be donated from patients who have been symptom-free for a minimum of two-weeks [19,32]. Nevertheless, there have been clinical trials in this space that have not yet been published (https://clinicaltrials.gov/ct2/show/NCT04373460). These efforts may lead to products manufactured from plasma donors, such as hyper-immune IgG, which can be a clinical product with greater control in treatment.

Monoclonal neutralizing antibodies are attracting attention as alternatives to CPT. With the development of humanized mouse antibodies and the subsequent generation of entirely human antibodies using various techniques, monoclonal antibodies have become widely implemented in therapy and prophylaxis for cancer, autoimmune diseases, and viral pathogens [21]. Indeed, a humanized mouse monoclonal antibody (palivizumab) neutralizing RSV is widely used prophylactically to protect vulnerable infants [33]. As mentioned in the previous section, the development of human B-cell isolation technology has facilitated the production of recombinant human antibodies. Recently, highly specific and often broadly acting neutralizing monoclonal antibodies have been developed against several viruses [21,34–37]. Passive immunization with a monoclonal antibody is being considered for treating COVID-19 caused by SARS-CoV-2 [22,38–41]. More than 20 neutralizing antibodies against SARS-CoV-2 have been used in clinical trials, several of which have been approved by the US FDA for emergency use by July 2021 (Table 1). An updated list of neutralizing antibodies in clinical studies can be found at the COVID-19 Biologics Tracker (https://www.antibodysociety.org/covid-19-biologics-tracker/). Including those not involved in clinical trials, over 80 monoclonal antibodies have been shown to neutralize pseudovirus or SARS-CoV-2 infection (reviewed in [6]).

REGN-COV2 is a cocktail of two monoclonal antibodies, designated as casirivimab (REGN10933) and imdevimab (REGN10987), which the FDA authorized for emergency use [42]. These two antibodies were isolated from a pool of neutralizing antibodies generated from humanized mice and convalescent patients. The antibodies simultaneously bind two distinct epitopes on the RBD of the spike protein. The use of this antibody cocktail aims to decrease the potential for virus escape mutants in response to selective pressure compared with a single-antibody treatment [43,44]. The efficacy of REGN-COV2 was evaluated in rhesus macaques and golden hamsters infected with SARS-CoV-2. Treatment with the antibody cocktail resulted in the reduction in viral load and severity of lung damage in both models when administered either prophylactically or therapeutically [45]. In addition, the treatment resulted in an effective reduction in viral load in infected patients when administered early [46].

**Figure 2.**
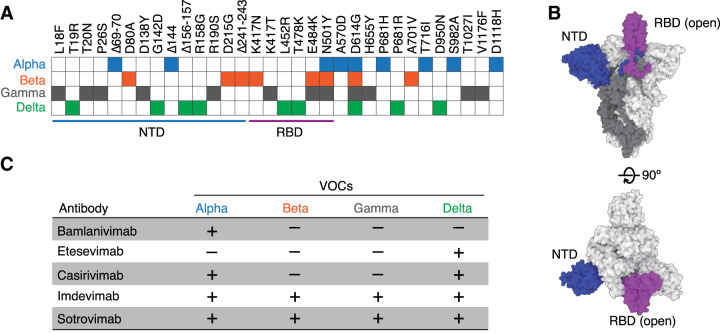
Variants of concern escape from antibodies in clinical use. (**A**) Amino acid substitutions in variants of concern (VOCs) mapped on spike protein [71]. (**B**) Cryo-EM structures of the spike protein (Protein Data Bank: 7DDN). Two orthogonal views, from the side and top, of the atomic model of the partially open spike trimer [93]. Blue and purple domains indicate the NTD and RBD (open), respectively. (**C**) Efficacy of neutralizing antibodies in clinical use against VOCs. This table summarizes the sensitivity of each monoclonal antibody to VOCs, but in practice, neutralizing antibodies are used in combination as a cocktail.

LY-CoV555, named bamlanivimab, is a potent anti-spike neutralizing antibody isolated from convalescent patients. Bamlanivimab binds to the RBD in the up (active) or down (resting) conformation, resulting in the inhibition of ACE2 binding. In a rhesus macaque model, the administration of bamlanivimab protected both the lower and upper airways from SARS-CoV-2 infection, supporting its antiviral efficacy [47]. LY-CoV016/CB6, designated as etesevimab, was also isolated from convalescent patients and inhibited infection with SARS-CoV-2 in rhesus macaques in both prophylactic and treatment settings [48]. Bamlanivimab monotherapy and a combination of bamlanivimab with etesevimab has emergency use authorization (EUA) for the therapeutic treatment of COVID-19 [49,50]. However, the FDA has revoked the EUA for bamlanivimab monotherapy on April 16, 2021, because some SARS-CoV-2 variants are resistant to bamlanivimab (see also section ‘variants of concern' and Figure 2C) [51].

VIR-7831 (also known as sotrovimab) is a derivative of S309, which was isolated from a convalescent patient recovered from SARS-CoV-1 in 2003 [52]. S309 potently neutralizes SARS-CoV-2 by engaging in the RBD of the spike protein. Sotrovimab has been engineered to improve the pharmacokinetic features and extend the half-life of S309 [20]. Sotrovimab received EUA from the US FDA for the treatment of COVID-19 patients [53].

PiN-21 is an ultrapotent homotrimeric nanobody identified from RBD-immunized llama serum using a proteomic strategy [54]. Nanobodies are minimal monomeric antigen-binding domains derived from camelid single-chain antibodies. Nanobodies can be administered by inhalation because they are small (∼15 kDa), highly soluble, and stable. In terms of cost, nanobodies are superior owing to their rapid production in bulk from microbes. Intranasal delivery of PiN-21 prevents and helps in treating SARS-CoV-2 infections in Syrian hamsters at ultra-low doses [55]. Combined with intranasal administration and lower production costs, PiN-21 could be a breakthrough treatment and prophylaxis.

## Vaccine-induced antibody

A variety of vaccines against SARS-CoV-2 have been used in practice, and many people have been vaccinated. Among them, mRNA vaccines were granted a historic first authorization for emergency use during the COVID-19 pandemic. This section focuses on antibodies induced by mRNA-based vaccines because of their flexibility and the potential of rapid production of any mRNA against future variants of SARS-CoV-2. The mRNA vaccines for SARS-CoV-2 are ∼95% effective in preventing COVID-19 [7–9]. Data on mRNA vaccine-induced immune responses in humans have been reported by some research groups. Turner et al. [56] generated 37 spike-binding monoclonal antibodies from three participants who received the Pfizer-BioNTech SARS-CoV-2 mRNA vaccine BNT162b2. Among them, 17 bound the RBD and six recognized the N-terminal domain (NTD). Notably, the three monoclonal antibodies showed cross-reactivity with the spike protein from the seasonal coronavirus OC43 or HKU1. These cross-reactive antibodies had higher levels of somatic hypermutation in their immunoglobulin heavy chain variable regions, suggesting a memory B cell origin. According to analyses by Amanat et al. [57] the polyclonal antibody response to mRNA-vaccination exceeds the titer observed in convalescent patients. However, the mRNA-vaccine induced more non-neutralizing antibodies than those found in COVID-19 survivors. They generated monoclonal antibodies from vaccinated individuals. These antibodies recognize the NTD, RBD, and S2 domains, whereas most vaccine antibodies do not have neutralizing activity *in vitro*. Among the small number of neutralizing antibodies, two antibodies target RBD, and five antibodies recognize NTD. The induction of cross-reactive antibodies to seasonal coronaviruses (OC43 and HKU1) was also confirmed. Although further studies are required to address the role of non-neutralizing antibodies induced by vaccines, a preprint study reported that a non-neutralizing antibody (CV3-13) obtained from a convalescent patient induced antibody-dependent cellular cytotoxicity, delayed neuroinvasion, and death in a transgenic K18-hACE2 mouse model [58].

Greaney et al. [59] investigated how human antibody responses to the mRNA-1273 (Moderna) vaccine are influenced by viral mutations. They conducted deep mutational scanning to compare the specificity of sera elicited by mRNA-1273 and natural infection with SARS-CoV-2. Vaccine-induced antibodies bind RBD more broadly across epitopes than infection-elicited antibodies. This suggests that single RBD mutations have a lesser impact on the neutralizing activity of vaccine-induced antibodies than that of infection-elicited antibodies. The neutralizing activity of sera from convalescent patients is relatively lower than that of vaccine-induced neutralizing activity [57], however, a single immunization to survivors boosted neutralizing titers against variants [60,61].

## Efficacy of monoclonal antibodies and vaccines against variants of concern

Multiple variants of SARS-CoV-2 have been reported, some of which are considered variants of concern (VOCs) due to their impact on public health [62]. VOCs are associated with increased infectivity and pathogenicity. As of July 2021, Alpha (B.1.1.7), Beta (B.1.351), Gamma (P.1), and Delta (B.1.617.2) variants were identified (Figure 2A,B). Of the four VOCs, the Delta variant has been predominant worldwide since it first appeared in India at the end of 2020 (https://ourworldindata.org/covid-vaccinations). Public Health England reported that Delta is associated with a 64% increased transmission compared with Alpha [63], which is 43 to 90% more transmissible than the predecessor lineage [64]. In addition, secondary attack rates for contacts of cases with Delta and no travel history are higher than those for contacts of non-travel cases with Alpha, 12.4% compared with 8.2% [65]. A preprint study reported that individuals infected with the Delta variant had viral loads averaging ∼1000 times greater (PCR Ct: 24.00) than those in patients infected with the original Wuhan Hu-1 strain (clade 19A/19B, PCR Ct: 34.31), suggesting potentially faster viral replication and higher infectiousness of the Delta variant during early infection [66]. The P681R mutation of the spike protein of Delta is located near the S1–S2 cleavage site (R685/S686) of furin (Figure 2A). A furin-cleaved product, the S2 region, drives the fusion of viral and host cell membranes. Alpha is the first variant to be spread worldwide. Like Delta, it also had a P681 change, but P681H (Figure 2A). The mutation may have increased replication, leading to higher viral loads and increased transmission [67]. The emergence of these new variant strains of SARS-CoV-2 threatens the treatment of the disease by passive immunization and prevention of infection with vaccines. Real-time data on SARS-CoV-2 mutants are available on the GISAID COVID-19 variant tracker (https://www.gisaid.org/hcov19-variants/).

Starr et al. [68] reported a complete map of SARS-CoV-2 RBD mutations that escaped recognition by bamlanivimab and its cocktail with etesevimab. Circulating VOCs with E484K mutation, Beta and Gamma, are sensitive to bamlanivimab. The cocktail of bamlanivimab and etesevimab did not prove to be effective against the combination of mutations at K417N/T and E484K in the Beta and Gamma variants. Other *in vitro* and *in vivo* studies reported that the Beta variants exhibited resistance to neutralization by a cocktail of bamlanivimab and etesevimab (Figure 2C) [69,70]. The Alpha variant escapes recognition by etesevimab but not by bamlanivimab *in vitro* [71]. Unfortunately, the Gamma variant escapes from bamlanivimab and etesevimab both [72]. Bamlanivimab lost antiviral activity against the Delta variant harboring the L452R mutation, while etesevimab retained neutralizing activity against the Delta variant [68,71]. On April 16, 2021, the FDA revoked the EUA for bamlanivimab monotherapy because of the sustained increase in variants resistant to bamlanivimab [51].

A prospective mapping of viral mutations on RBD shows that casirivimab and imdevimab are evaded owing to a largely non-overlapping set of mutations, respectively [73]. This is consistent with the report that these antibodies target distinct epitopes based on structural analysis [44]. The cocktail of casirivimab and imdevimab reduced the escape fraction on the map as expected, whereas recognition by the cocktail was evaded owing to the E406W mutation. Even though the mutation is not present among circulating SARS-CoV-2 to date, a virus with this mutation would reduce the efficacy of REGN-COV2. As Beta and Gamma are already resistant to neutralization by casirivimab, the mutant viruses are to be a threat to treatment with REGN-COV2 (Figure 2C) [69–71]. Both casirivimab and imdevimab remained active against Alpha and Delta variants [71]. Together, the cocktail of casirivimab and imdevimab, termed REGN-COV2, provides protection against all four VOCs. In addition, the triple combination of non-competing antibodies (casirivimab, imdevimab, and REGN10985) limits the emergence of new escape variants *in vitro* [74]. In the ongoing emergence of SARS-CoV2 variants, it has become apparent that some monoclonal antibodies lose their neutralizing efficacy. Of the neutralizing monoclonal antibodies authorized by the FDA, sotrovimab (also known as VIR-7831, S309) and imdevimab have shown neutralizing activity *in vitro* against all four VOCs (Figure 2C) [70,75]. However, with the emergence of new variants, these two antibodies may lose their effectiveness.

There are examples of other viruses, such as RSV, HIV, and Ebola, where the use of monoclonal antibodies has been shown to be associated with prevention or treatment failure [76–78]. In the case of RSV, suptavumab can bind and block RSV type A infection in preterm infants, while RSV type B has a two-amino acid substitution in the suptavumab epitope that leads to a loss of neutralization activity. Although previously circulating RSV type B strains lacked these mutations and were therefore effectively neutralized by suptavumab, the predominant circulating RSV type B lineage at the time of the trial had the two-point mutations rendering resistance to the monoclonal antibody [76]. This clearly illustrates the dangers of antibody monotherapy. It might be possible to maintain the efficacy of antibody therapy by identifying viral variants in patients and administering a combination of neutralizing antibodies. Figure 2C summarizes the sensitivity of each monoclonal antibody to VOCs, but in practice, the neutralizing antibodies are used in combination (https://www.covid19treatmentguidelines.nih.gov/tables/table-a/). Continuous development of cross-neutralizing antibodies is also required against newly arising variants [79–81].

The efficacy of vaccines against emerging variants has also been investigated. The plasma neutralizing activities of volunteers who were administered mRNA-vaccines (Moderna and Pfizer) were equivalent to those of survivors. However, the vaccine-elicited activities were reduced against SARS-CoV-2 pseudotype virus variants harboring E484K, N501Y, and K417N/E484K/N501Y mutations, respectively, which were found in Alpha, Beta, and Gamma variants [82] (Figure 2A). The monoclonal antibodies generated from the volunteers showed potent neutralizing activities, as well as monoclonal antibodies elicited from natural infections. However, 14 of the 17 most potent antibodies were reduced or abolished by the K417N, E484K, or N501Y mutations. According to a comprehensive analysis of plasma neutralization processes using 16 authentic variants, a range of reduction in the neutralization capacity is associated with the E484K, N501Y/T, and L452R mutations [83]. Another study reported that sera from recipients receiving two doses of Pfizer or AstraZeneca vaccines were less potent against Beta and Delta variants, relative to Alpha [71]. Notably, sera from individuals who received one dose of vaccine barely inhibited variants, while two doses elicited cross-neutralizing activities against the VOCs, albeit weaker. It was also reported that a second dose of mRNA-vaccine elicited neutralization activity against all four VOCs [84–86]. In addition, sera from convalescent patients collected 12 months after symptoms lost their neutralizing activity, whereas the convalescent patients acquired cross-neutralizing activity against VOCs after one dose of vaccination [71]. Similar results have been reported by two research groups. Single-dose vaccination to recovered donors confers enhanced T cell immunity and memory B cell response, resulting in boosted neutralizing titers against variants [60,61]. Low-dose of mRNA-vaccine generates durable memory enhanced by cross-reactive T cells [87]. These reports show that T-cell mediated immunity and immune memory are important clinical effects of vaccines. Evidence supports the importance of neutralizing antibodies, demonstrating a relationship between neutralizing antibody levels and breakthrough infections [88]. Despite this evidence, vaccine-induced neutralizing antibodies are likely not the only mechanism of their clinical effectiveness. The Pfizer-BioNTech vaccine induced a germinal center response sustained by memory B cells and the T follicular helper cell response, producing antibodies with cross-reactivity to the spike protein from the seasonal coronaviruses OC43 and HKU1 [56]. Early induction of T cell-mediated immunity also plays a significant role in viral clearance and mild disease [89]. Importantly, T cells from exposed donors or those activated by vaccinees effectively recognize SARS-CoV-2 variants [90,91]. Therefore, a decrease in neutralizing activity *in vitro* does not predict that vaccines will be ineffective. T cell-mediated immunity is likely to protect vaccinated individuals from the severe disease in the near future, regardless of the identity of SARS-CoV-2 variants in circulation [92]. Indeed, a Public Health England clinical study published in July 2021 reported vaccine effectiveness against symptomatic disease from the Alpha and the Delta variants [67]. Effectiveness after a single dose of vaccine was notably lower against the Delta variant (30.7%) than the Alpha variant (48.7%). However, the second dose of Pfizer mRNA-vaccine (BNT162b2) boosted protection against Delta to 88.0% compared with 93.7% against Alpha. Although the Delta variant is moderately resistant to vaccines, a high level of effectiveness against Delta was confirmed after the administration of two doses in clinical trials. This suggests that two vaccine doses induce a germinal center response sustained by memory B cells and T helper cells, resulting in cross-reactivity to different variants. While the emergence of variants could reduce the efficacy of vaccines against symptoms, vaccine-induced T cell immunity could be conserved in terms of preventing severe disease against currently circulating variants in the near future. However, the further development of vaccines combined with careful variant monitoring is required for future infection control [81].

## Conclusions

In the hope of treatment and prevention of COVID-19, the rapid development of neutralizing antibodies against SARS-CoV-2 from convalescent patients has been driven by a combination of single-cell cloning and deep-sequencing technologies. To end the pandemic, COVID-19 vaccines have been developed and distributed at an unprecedented pace. The emergence of new variants of SARS-CoV-2 has affected the treatment of the disease with neutralizing antibodies and the effectiveness of vaccines. However, even if neutralizing activity reduces after vaccination, vaccine-induced T cells and memory B cells could prevent vaccinated individuals from severe disease against currently circulating variants, including VOCs, variants of interest, variants under monitoring (https://www.who.int/en/activities/tracking-SARS-CoV-2-variants/). It is also hoped that vaccine design and administration methods can efficiently induce a germinal center response. To keep pace against severely pathogenic variants, neutralizing antibodies and vaccines should be examined periodically to determine their efficacy against emerging variants. Continued surveillance of variants is required for the screening of neutralizing antibodies and the design of vaccines in the future.

## Perspectives

- Treatment with neutralizing antibodies and prophylaxis with vaccines have been shown to be effective against COVID-19; however, the emergence of virus mutants threatens their effectiveness.
- The development of single-cell isolation and sequencing technologies has facilitated the isolation of neutralizing antibodies. In addition, the practical application of mRNA-based vaccines has made it easier to design vaccines for emerging variants.
- Continuous development of neutralizing antibodies and vaccines is required to deal with the newly arising virus variants.

## Abbreviations

ACE2: angiotensin-converting enzyme 2
AIRR: Adaptive Immune Receptor Repertoire
COVID-19: coronavirus disease
CPT: convalescent plasma therapy
ELISA: enzyme-linked immunosorbent assay
EUA: Emergency Use Authorization
FcγR: Fc gamma receptor
FDA: US Food and Drug Administration
MERS: Middle East respiratory syndrome
NTD: N-terminal domain
RBD: receptor-binding domain
RSV: respiratory syncytial virus
SARS-CoV-2: severe acute respiratory syndrome coronavirus 2
VOCs: variants of concern
